# Interplay Between n-3 and n-6 Long-Chain Polyunsaturated Fatty Acids and the Endocannabinoid System in Brain Protection and Repair

**DOI:** 10.1007/s11745-017-4292-8

**Published:** 2017-09-05

**Authors:** Simon C. Dyall

**Affiliations:** Faculty of Health and Social Sciences, Bournemouth University, Dorset, UK

**Keywords:** Endocannabinoid system, Neurogenesis, Neuroinflammation, Omega-3 fatty acids, Omega-6 fatty acids

## Abstract

The brain is enriched in arachidonic acid (ARA) and docosahexaenoic acid (DHA), long-chain polyunsaturated fatty acids (LCPUFAs) of the n-6 and n-3 series, respectively. Both are essential for optimal brain development and function. Dietary enrichment with DHA and other long-chain n-3 PUFA, such as eicosapentaenoic acid (EPA), has shown beneficial effects on learning and memory, neuroinflammatory processes, and synaptic plasticity and neurogenesis. ARA, DHA and EPA are precursors to a diverse repertoire of bioactive lipid mediators, including endocannabinoids. The endocannabinoid system comprises cannabinoid receptors, their endogenous ligands, the endocannabinoids, and their biosynthetic and degradation enzymes. Anandamide (AEA) and 2-arachidonoylglycerol (2-AG) are the most widely studied endocannabinoids and are both derived from phospholipid-bound ARA. The endocannabinoid system also has well-established roles in neuroinflammation, synaptic plasticity and neurogenesis, suggesting an overlap in the neuroprotective effects observed with these different classes of lipids. Indeed, growing evidence suggests a complex interplay between n-3 and n-6 LCPUFA and the endocannabinoid system. For example, long-term DHA and EPA supplementation reduces AEA and 2-AG levels, with reciprocal increases in levels of the analogous endocannabinoid-like DHA and EPA-derived molecules. This review summarises current evidence of this interplay and discusses the therapeutic potential for brain protection and repair.

## Introduction

N-6 and n-3 long-chain polyunsaturated fatty acids (LCPUFA) are essential components of membrane phospholipids and also precursors to a large and ever expanding repertoire of bioactive lipid mediators. The brain is highly enriched in the n-6 PUFA, arachidonic acid (ARA), and the n-3 PUFA, docosahexaenoic acid (DHA), with both essential for optimum brain development and function [1]. Elevated dietary intake of DHA and eicosapentaenoic acid (EPA), another n-3 LCPUFA, has beneficial effects on learning and memory, decreases neuroinflammatory processes and enhances synaptic plasticity and neurogenesis [2]. Similarly, inverse relationships are typically observed between fish intake or blood DHA levels and age-related cognitive decline [3]. However, recent estimates indicate that worldwide many populations are currently consuming DHA and EPA at levels well below the recommendations issued by many international authorities [4–6].

The mode of action of the LCPUFA is still poorly understood and is further complicated by the diverse repertoire of bioactive lipid mediators that can be generated. For example, ARA is the precursor to a wide range of mediators, including the two major endocannabinoids in the brain [7]. The endocannabinoid system has similarly been shown to have important roles in neuroprotective and pro-neurogenic processes, such as attenuating chronic neuroinflammation, regulating pro-inflammatory cytokine release and enhancing synaptic plasticity and adult neurogenesis [8, 9], and importantly has shown therapeutic potential in brain ageing and neurodegenerative conditions [10].

Thus, there is considerable overlap in effects of n-3 PUFA and the endocannabinoid system; however, these different classes of lipid mediators have traditionally been viewed and researched separately. This view is now being challenged as there are a growing number of independent lines of evidence suggesting a complex interplay between them. For example, analogous series of ethanolamide endocannabinoid-like molecules derived from DHA and EPA have been identified, although their biological roles have yet to be established [11, 12]. Furthermore, in recent elegant work long-term dietary n-3 PUFA deficiency in mice abolished endocannabinoid-mediated neuronal functions across a range of different brain regions, showing for the first time how the endocannabinoid system can be regulated by manipulation of the dietary n-6:n-3 PUFA ratio [13–15]. This is a cause for concern as the Western diet typically has an n-6:n-3 PUFA ratio of around 15:1, whereas the ideal ratio is thought to be closer to 4:1 [16]. This unbalanced intake is reflected in low to very low tissue levels of DHA and EPA [17], and may also be involved in the aetiology of many diseases, such as cardiovascular disease, cancer, inflammatory and autoimmune diseases [16].

The aim of this review is to summarise current evidence of the interplay between n-3 and n-6 LCPUFA and the endocannabinoid system and discusses the potential role of modifying their levels through dietary manipulation of n-6 and n-3 PUFA intake with the aim of ameliorating neuroinflammation and enhancing brain protection and repair, particularly in ageing.

## Metabolism of PUFA and Endocannabinoids

**Fig. 1:**
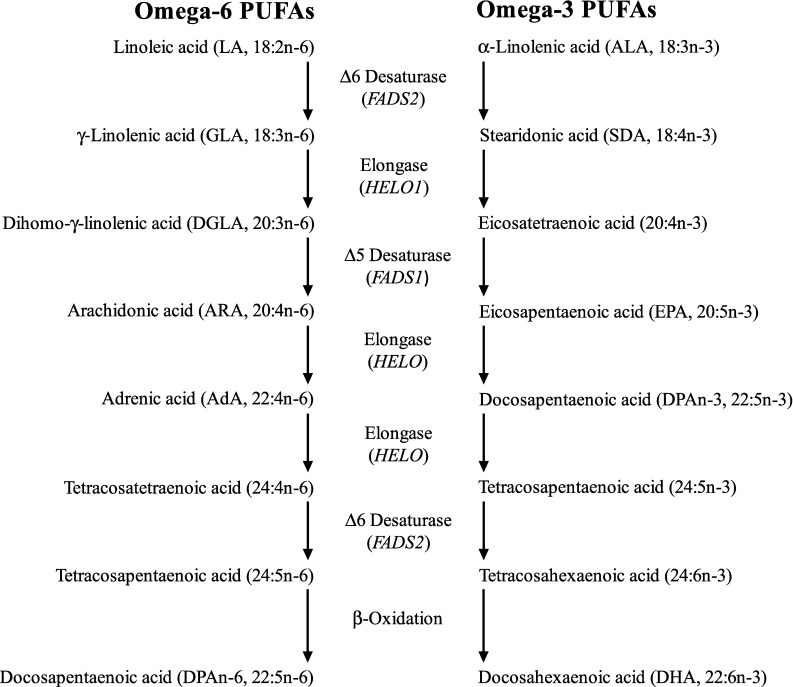
N-6 and n-3 PUFA metabolism and lipid mediators produced from ARA, DHA and EPA. Synthesis of n-6 and n-3 LCPUFA begins with desaturation of LA and ALA to γ-linolenic acid (GLA, 18:3n-6) and stearidonic acid (18:4n-4), respectively, catalysed by Δ6 desaturase (*FADS2* gene). GLA is elongated to dihomo-γ-linolenic acid (DGLA, 20:3n-6) and SDA to eicosatetraenoic acid (20:4n-3) (*ELOVL1* gene). Δ5-Desaturase (*FADS1* gene) converts DGLA to ARA (20:4n-6) and 20:4n-3 to EPA (timnodonic acid, 20:5n-3). Two cycles of elongation (elongase-2, *ELOVL2* gene) convert ARA to adrenic acid (AdA, 22:4n-6) and then tetracosatetraenoic acid (24:4n-6), and EPA to docosapentaenoic acid (DPAn-3, clupanodonic acid, 22:5n-3) and then tetracosapentaenoic acid (24:5n-3). A second desaturation by Δ6 desaturase produces tetracosapentaenoic acid (24:5n-6) and tetracosahexaenoic acid (nisinic acid, 24:6n-3), respectively. These are translocated to the peroxisome for β-oxidation by acyl-coenzyme-A oxidase (*ACOX1* gene) and d-bifunctional enzyme (*HSD1784* gene) and peroxisomal thiolases to produce docosapentaenoic acid (DPAn-6, osbond acid, 22:5n-6) and DHA (cervonic acid, 22:6n-3), which are translocated back to the endoplasmic reticulum

ARA and DHA are the two main PUFA in the brain [2]. These LCPUFA can be supplied either preformed from the diet or synthesised in the liver from their shorter chain precursors, linoleic acid (LA, 18:2n-6) and α-linolenic acid (ALA, 18:3n-3), respectively [18, 19]. However, the efficiency of conversion in humans is extremely limited [20], and due to the shared nature of the biosynthetic pathways, imbalances in the dietary intake of LA and ALA will result in reciprocal inhibition of the opposing pathway and further limit conversion [21]. Therefore, the most efficient way to increase tissue levels of LCPUFA is by intake of the preformed LCPUFA. The n-6 and n-3 PUFA biosynthetic pathways are shown in detail in Fig. 1.

**Fig. 2:**
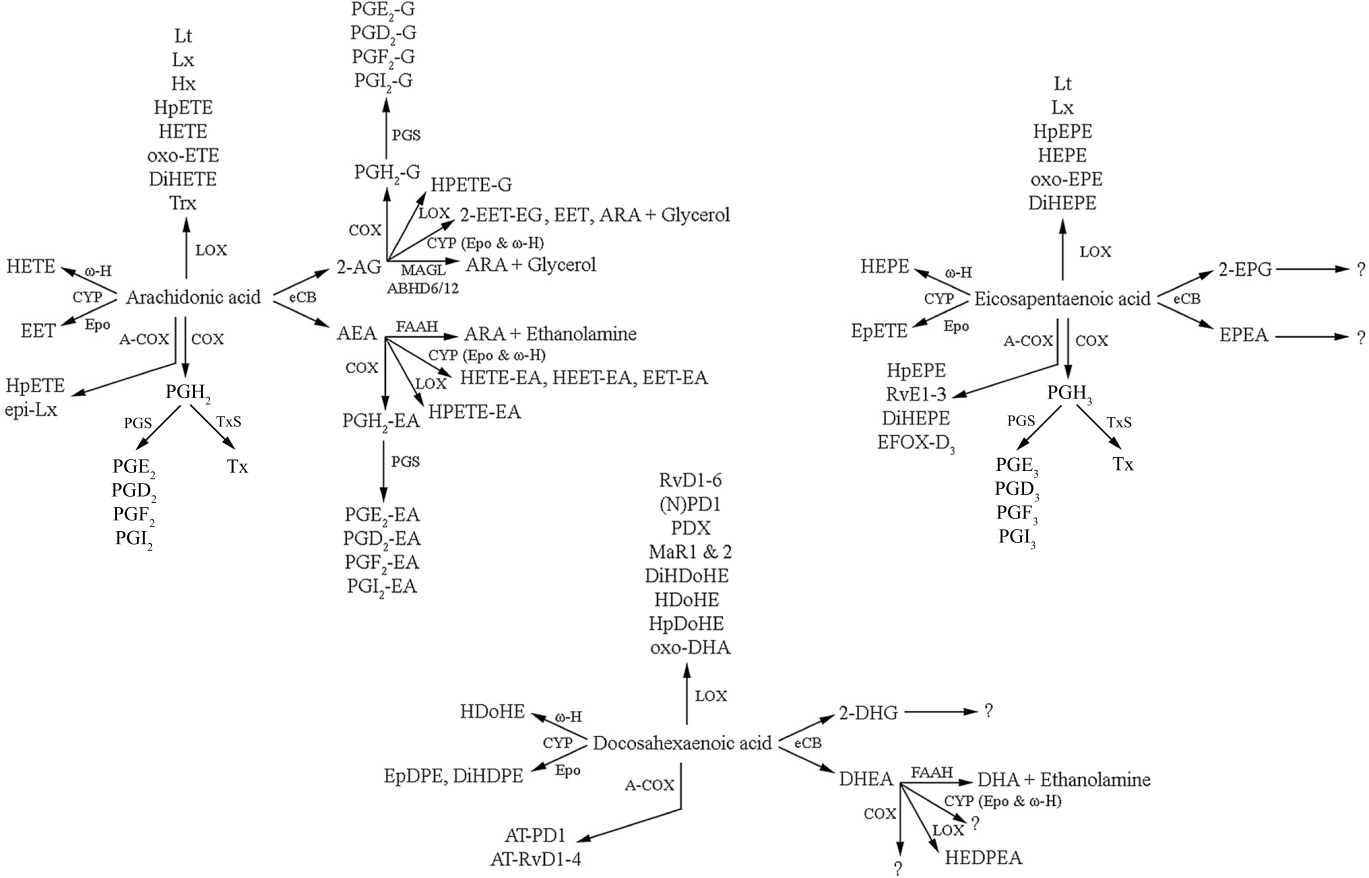
Main lipid mediators produced from ARA, DHA and EPA. ARA, DHA and EPA are precursors to multiple metabolites, including oxylipins produced by cyclooxygenase (COX) and acetylated COX-2 (A-COX), lipoxygenase (LOX) and cytochrome P450 (CYP) enzymes and the endocannabinoids (eCB). The major pathways in the synthesis of ARA, DHA and EPA-derived endocannabinoids are shown in Fig. 3. *2-AG* 2-arachidonoylglycerol, *2-DHG* 2-docosahexaenoylglycerol, *2-EET-EG* 2-epoxy-eicosatrienoic acid glycerol, *2-EPG* 2-eicosapentaenoylglycerol, *ABHD6/12* α/β-Hydrolase domain containing 6 or 12, *AEA N*-arachidonoylethanolamide (anandamide), *AT* aspirin-triggered, *DHEA N*-docosahexanoylethanolamine (synaptamide), *DiHDoHE* dihydroxy-docosahexaenoic acid, *DiHDPE* dihydroxy-docosapentaenoic acid, *DiHEPE* dihydroxy-eicosapentaenoic acid, *DiHETE* dihydroxy-eicosatetraenoic acid, *DiHETrE* dihydroxy-eicosatrienoic acid, *EDP* epoxy-docosapentaenoic acids, *EET* epoxy-eicosatrienoic acid, *EET-EA* epoxy-eicosatrienoic acid ethanolamide, *EETeTr* epoxy-eicosatetraenoic acids, *EFOX* electrophilic fatty acid oxo-derivatives, *EpDPE* epoxy-docosapentaenoic acid, *EPEA N*-eicosapentaenoylethanolamine, *EpETE* epoxy-eicosapentaenoic acid, *EpETrE* epoxy-eicosatrienoic acid, *Epo* epoxygenase, *FAAH* fatty acid amide hydrolase, *HDoHE* hydroxy-docosahexaenoic acid, *HEDPEA* hydroxy-epoxy-docosapentaenoyl ethanolamide, *HEET-EA* hydroxyepoxy-eicosatrienoic acid ethanolamide, *HEPE* hydroxy-eicosapentaenoic acid, *HETE* hydroxy-eicosatetraenoic acid, *HETE-EA* hydroxy-eicosatetraenoic acid ethanolamide, *HHTrE* hydroxy-heptadecatrienoic acid, *HpDoHE* hydroperoxy-docosahexaenoic acid, *HpEPE* hydroperoxy-eicosapentaenoic acid, *HpETE* hydroperoxy-eicosatetraenoic acid, *Hx* hepoxilin, *Lt* leukotriene, *Lx* lipoxin, *MAGL* monoacylglycerol lipase, *MaR* maresin, *(N)PD1* (neuro)protection D1, *oxo-EET* oxo-eicosatetraenoic acid, *PGD* prostaglandin D metabolite, *PGE* prostaglandin E metabolite, *PGF* prostaglandin F metabolite, *PGI* prostacyclin, *PGS* prostaglandin E, D or F or prostacyclin synthase, *PD* protectin, *RvD* resolvin D series, *RvE* resolvin E series, *Tx* thromboxane, *TxS* thromboxane synthase, *Trx* trioxilin, from DHA and hydroxy-eicosapentaenoic ϖ-hydrolase

Endogenous synthesis of LCPUFA is low within the brain compared with uptake from the unesterified plasma fatty acid pool [22, 23], suggesting brain levels are maintained via uptake from dietary and/or liver sources in blood. Although LCPUFAs appear to cross the blood-brain barrier via simple diffusion [24], active transporters have been identified that may play a role in regulating the specificity of LCPUFA concentrations [20]. Further multiple mechanisms including β-oxidation, decreased incorporation, elongation and lower phospholipid recycling have also been identified, which maintain the high ARA and DHA concentration in relation to other LCPUFAs [25, 26]. However, brain LCPUFA composition is responsive to dietary intake, such that a diet high in LA, with an LA:an ALA ratio of 10:1 typical of a Western diet decreases brain DHA accretion and increases adrenic acid (AdA, docosatetraenoic acid, 22:4n-6) and docosapentaenoic acid (DPAn-6, 22:5n-6) levels [27], whereas a diet with an LA:ALA ratio of 1:1, more similar to that encountered during our evolution [16], leads to higher brain DHA levels. Imbalances in intake not only compromise brain LCPUFA content, but may also impact on the production of a wide range of mediators derived from these LCPUFA, thereby potentially negatively influencing brain activity and function.

The fatty acid composition of neuronal membranes influences cellular function through direct effects on membrane biophysical properties, but also by providing a precursor pool for signalling molecules and lipid-derived mediators [1]. N-6 and n-3 LCPUFA are the precursors to a vast array of bioactive mediators involved in many cellular processes, particularly related to the inflammatory response [2]. Three main pathways are involved in the production of these oxylipin mediators: (1) cyclooxygenase (COX, also known as prostaglandin endoperoxide H synthase or PGHS) and subsequent synthases, (2) lipoxygenase (LOX) and (3) cytochrome P450 mixed function oxidase enzymes (CYP) [28]. These canonical pathways produce the classic mediators, with those produced from C20 PUFA, such as ARA and EPA, called eicosanoids, whereas those from C22 PUFA, such as DHA, are called docosanoids. Analogous series of oxylipins generated from LA, dihomo-γ-linolenic acid (DGLA), AdA and ALA and the n-3 docosapentaenoic acid (DPAn-3) have also been identified, but their roles are not well characterised in the literature and are therefore not the focus of this review. However, the interested reader is referred to an excellent review by Gabbs and colleagues [29].

COX catalyses the initial oxygenation of non-esterified fatty acids to produce prostaglandin H (PGH), a short-lived intermediate, which is further metabolised into prostanoids, such as other prostaglandin series (PGD, PGE, PGF), prostacyclins (PGI), thromboxanes (Tx), and lipoxins (Lx), hydroxy and hydroperoxy fatty acids [30]. Vertebrates have two principal isoforms of COX: COX-1 and COX-2 [31]. COX-1 is constitutively expressed, whereas although COX-2 is an inducible enzyme in most tissues, in the cortex, hippocampus and amygdala constitutive expression is observed [32, 33]. COX-2 is not only a key enzyme in the inflammatory and neuroinflammatory processes, but has important roles in the regulation of neural activity, such as learning and memory [34]. COX-2 oxygenates a wide range of fatty acids and fatty esters [35].

COX-2 was traditionally thought to be responsible for causing inflammation and neuroinflammation by converting ARA to PG and Tx; however, this simplified model has been reconsidered with a greater understanding of the delicate balance between positive and negative feedback loops [36]. For example, PGE_2_ and PGD_2_ are pro-inflammatory mediators responsible for the induction of inflammation, but at a later stage in the process are also responsible for class switching of eicosanoid production from PG and leukotrienes (Lt) to Lx [36]. It has consistently been shown that increasing dietary n-3 PUFA changes the lipid profile of membranes and alters the balance of n-6 and n-3 PUFA competing as substrates for COX, consequently altering the series of prostaglandins synthesised, which ultimately alters cellular responses to mitogenic and inflammatory stimuli [37–41]. This has been demonstrated in many cells throughout the body, including glial cells [42].

LOX catalyse the formation of hydroxyl fatty acids and their metabolites, such as Lt, Lx and the “specialised lipid mediators” (SPM) [29]. These included the resolvins (Rv), protectins (PD) and maresins (MaR) derived from n-3 LCPUFA [43]. LOX enzymes are traditionally classified based on the position of the hydroxyl and hydroperoxy fatty acids they produce from ARA, e.g. 5-LOX forms 5-hydroxy-eicosatetraenoic acid (5-HETE) and 5-hydroperoxy-eicosateraenoic acid (5-HpETE); however, this system has limitations as the position varies according to different chain lengths of the substrates and some LOX act at more than one position [29].

The SPMs are a rapidly expanding class of molecules involved in the active resolution of inflammation produced via COX and LOX catalysed pathways [43]. D-series resolvins (RvD), PD and MaR are from produced from DHA, whereas E-series resolvins (RvE) are from EPA [44]. A further series of RvD and MaR has recently been identified generated from DPAn-3, including RvD1_n-3 DPA_ and MaR1_n-3 DPA_, which demonstrate similar anti-inflammatory and pro-resolving properties to those from DHA and EPA [45, 46]. The SPMs act via a series of cell-type specific receptors, for example, RvD1 binds GPR32 and lipoxin A_4_ receptor (ALx), and RvE1 binds the ChemR23 orphan receptor and leukotriene B_4_ receptor (BLT_1_) [47]. The best characterised SPM in terms of nervous system protection is (neuro)protectin D1 (NPD1, 10*R*-17*S*-dihydroxy-docosahexaenoic acid), which is biosynthesised in response to injury and may have therapeutic potential in a wide range of neurological conditions [48, 49]. In addition, acetylation of COX-2 by aspirin blocks PG biosynthesis, but COX-2 is still able to produce HETE from ARA, hydroxy-docosahexaenoic acid (HDoHE) from DHA and hydroxy-eicosapentaenoic acid (HEPE) from EPA, which can be transformed by leukocytes to aspirin-triggered forms of Lx, Rv and PD [50].

A further class of metabolites generated from n-3 PUFA by LOX is the electrophilic fatty acid oxo-derivatives (EFOX), with 7-oxo-DHA 7-oxo-DPA and 5-oxo-EPA produced from DHA, DPAn-3 and EPA, respectively [51, 52]. EFOXs display a wide range of anti-inflammatory actions, including acting as agonists nuclear receptors, such as the peroxisome proliferator-activated receptor (PPAR) and inhibiting cytokine production in activated macrophages [52]. Furthermore, consistent with the formation of aspirin-triggered SPM, acetylation of COX-2 by aspirin also significantly increases the formation of EFOX [2].

The third oxidative pathway involves CYP epoxygenases and ϖ-hydrolases, which metabolise PUFA to lipid mediators with many diverse biological functions at both the systemic and cellular levels [53, 54]. Regio- and stereoisomers of epoxy-eicosatetraenoic acids (EET) and HETE are produced from ARA, whereas those derived from EPA include epoxy-eicosatetraenoic acids (EETeTR) and hydroxy-eicosapentaenoic acids (HEPE) and epoxy-docosapentaenoic acids (EDP) and HDoHE from DHA [54]. EPA is the preferred substrate for most isoforms of CYP, with metabolism of DHA and ARA occurring at similar rates [54]. Expression of CYP isoforms occurs in multiple cell types across the brain, including astrocytes, neurons and endothelial cells [53].

In addition, n-6 and n-3 PUFAs are also precursors to endogenous ligands of the endocannabinoid receptors (endocannabinoids). The endocannabinoid system is made up of the cannabinoid receptors (CB1 and CB2 receptors), endocannabinoids and the enzymes required for endocannabinoid synthesis and degradation [55]. Two families of endocannabinoids have been identified, 2-acylglycerols and ethanolamides; however, not all congeners are ligands of the cannabinoid receptors [56]. The most abundant and best characterised endocannabinoids in the brain are the 2-acylglycerol, 2-arachidonoylglycerol (2-AG) and the ethanolamide, *N*-arachidonoylethanolamine (AEA, anandamide), which are both derived from ARA [7]. Further n-6 PUFA-derived endocannabinoids include dihomo-γ-linolenoyl ethanolamide, docosatetraenoyl ethanolamide, 2-arachidonyl glycerol ether (noladin ether), *O*-arachidonoylethanolamine (virodhamine) and *N*-arachidonoyldopamine; however, although these endocannabinoids can bind to cannabinoid receptors, their function is still unclear and will not therefore be discussed further in this review [57]. Analogous series of endocannabinoids have been identified from n-3 PUFA. Alpha-linolenoylethanolamide (ALEA) is produced from ALA and has been identified in human plasma, where levels were shown to be responsive to dietary ALA supplementation [58]. However, the best characterised n-3 PUFA-derived endocannabinoids are produced from DHA and EPA, with the 2-acylglycerols, 2-docosahexaenoylglycerol (2-DHG) and 2-eicosapentaenoylglycerol (EPG), and the ethanolamides, *N*-docosahexaenoylethanolamine (DHEA) and *N*-eicosapentaenoylethanolamine (EPEA), generated from DHA and EPA, respectively [12, 59]. This review will focus on the endocannabinoids derived from ARA, DHA and EPA.

**Fig. 3:**
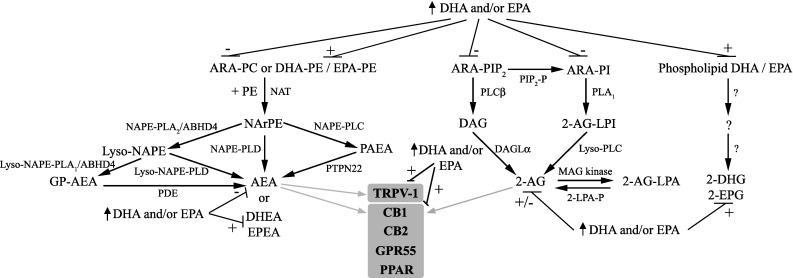
Interplay in the synthesis and actions of the 2-acylglycerols and ethanolamides derived from ARA, DHA and EPA. The major pathway for AEA production begins with *N*-acyltransferase (NAT) transferring ARA from phosphatidylcholine (ARA-PC) to phosphatidylethanolamine (PE) to generate *N*-arachidonoyl phosphatidylethanolamine (NArPE), which is followed by hydrolysis by *N*-acyl phosphatidylethanolamine-selective phospholipase D (NAPE-PLD) to produce AEA. Further pathways include NAPE deacylation by the α/β-hydrolase domain containing 4 (ABHD4) and either the glycerophosphoarachidonoylethanolamide produced (GP-NAPE) cleaved by phosphodiesterase (PDE) to produce AEA or lyso-NAPE is hydrolysed by lyso-NAPE-phospholipase D (PLD) directly to AEA. NAPE can also be hydrolysed by phospholipase C (NAPE-PLC) to generate phospho-anandamide (PAEA), which is dephosphorylated to AEA by phosphatases such as protein tyrosine phosphatase (PTPN22). DHEA and EPEA production from phospholipid bound DHA and EPA appears to share the same pathways. Synthesis of 2-AG occurs from phosphatidylinositol-bound ARA (ARA-PI) via phospholipase C-β (PLCβ) and production of an ARA-diacylglycerol (DAG), which is hydrolysed by diacylglycerol lipases-α to produce 2-AG. Further pathways include dephosphorylation of 2-AG-lysophosphatidic acid (2-AG-LPA) by LPA phosphatase (2-LPA-P) or via phospholipase A_1_ (PLA_1_) converting PI to 2-arachidonoyl-lyso PI (2-AG-LPI) and then to 2-AG by lyso phospholipase C (lyso-PLC). The pathways of 2-DPG and 2-EPG production are currently unknown. 2-AG and AEA act at CB1 and CB2 receptors, GPR55 and PPAR, with AEA additionally acting at TRPV-1 (shown in *grey*). Dietary DHA and EPA enrichment decreases phospholipid ARA and increases phospholipid DHA and EPA, and favours production of DHA and EPA-derived endocannabinoids, whereas acute DHA and EPA treatment *in vitro* increases 2-AG. DHA and EPA also regulate CB1, CB2 TRPV-1 and PPAR receptor activity and levels. For detailed explanations, refer to the text

AEA and 2-AG are produced from membrane-bound phospholipid ARA, with synthesis occurring at the post-synaptic terminal via increased levels of intracellular calcium with both made in response to demand and rapidly degraded to ARA or oxygenated to further bioactive mediators [60]. The major pathways for the biosynthesis and degradation of AEA and 2-AG are described below and summarised in Fig. 3. However, the exact nature of these pathways is still to be resolved because of the complexity of the endocannabinoid system and presence of multiple often redundant pathways [61].

AEA production occurs via a series of steps from the membrane phospholipid precursor, sn-1 ARA phosphatidylcholine [62]. A calcium-dependent *N*-acyltransferase (NAT) transfers ARA to the nitrogen atom of phosphatidylethanolamine (PE) to generate *N*-arachidonoyl phosphatidylethanolamine (NArPE), which is followed by hydrolysis by an *N*-acyl phosphatidylethanolamine-selective phospholipase D (NAPE-PLD) to produce AEA [63]. Further parallel pathways have been identified, whereby NAPE is deacylated by α/β-hydrolase domain containing 4 (ABHD4) and either the glycerophosphoarachidonoylethanolamide produced (GP-NAPE) cleaved by a metal-dependent phosphodiesterase (PDE) to produce AEA or lyso-NAPE is hydrolysed by lyso-NAPE-phospholipase D (PLD) directly to AEA. NAPE can also be hydrolysed by phospholipase C (NAPE-PLC) to generate phospho-anandamide (PAEA), which is dephosphorylated by phosphatases such as protein tyrosine phosphatase 22 (PTPN22) to AEA [63]. Studies with NAPE-PLD knock-out mice indicate that NAPE-PLD is the major pathway for NAPE hydrolysis; however, the formation of AEA in the brain readily occurs via NAPE-PLD-independent pathways [64, 65].

The major pathway for the synthesis of 2-AG in the brain occurs from phosphatidylinositol (PI)-bound ARA via phospholipase C-β (PLCβ), which produces sn-1-acyl-2-arachidonoylglycerol, an ARA-diacylglycerol (DAG) [66]. DAG is then hydrolysed into 2-AG by the action of diacylglycerol lipases-α or -β (DAGL-α or DAGL-β), with the removal of the acyl group [66]. DAGLα appears to be the main isoform for 2-AG formation in the brain, as basal and stimulus-induced 2-AG content of the brain is greatly reduced in DAGLα, but not DAGLβ knock-out mice. [67]. Further pathways for the synthesis of 2-AG include dephosphorylation of 2-AG-lysophosphatidic acid (2-AG-LPA) by an LPA phosphatase (2-LPA-P) or via the sequential action of PLA_1_ converting PI to 2-arachidonoyl-lyso PI (2-AG-LPI) and then to 2-AG by lyso phospholipase C (lyso-PLC) [66].

DHEA and EPEA appear to be produced by the same biosynthetic pathways as AEA [68], whereas the synthesis of 2-DHG and 2-EPG is not well characterised in the literature. However, it is likely they are produced via the same pathways as 2-AG, as chronic DHA and EPA supplementation reduces 2-AG and AEA levels across a range of tissues including the brain, with reciprocal increases in levels of DHEA and 2-DHG, and 2-EPG [12, 69–72]. These alterations suggest competition for shared biosynthetic pathways as DHA and EPA displace ARA from membrane phospholipids. Interestingly, recent work in our laboratory found that acute administration of DHA or EPA significantly increased 2-AG, although not AEA levels in neural stem cells [73]. This increase may be driven by competition for the inactivating enzymes, such as COX-2, although further work is needed to fully elucidate the underlying mechanisms.

AEA and 2-AG predominantly act at the guanine-nucleotide-binding protein (G protein)-coupled receptor (GPCR) cannabinoid receptors, CB1 and CB2 [74]. The CB1 receptor is widely expressed in the brain, where it is the most abundant GPCR, highly expressed in the cortex, hippocampus, cerebellum and basal ganglia [74]. CB2 receptors were initially identified in cells of the immune system [75], but more recently have additionally been described in glia and subsets of neurons in the brain [76]. In addition, AEA and 2-AG have also been shown to act at the orphan receptor, GPR55 [77], and peroxisome proliferator-activated receptors (PPAR) [78]. PPARs are nuclear acting transcription factors with three subtypes, α, β (δ) and γ, and are involved in many cellular processes; for example, PPARγ regulates genes involved in neuroinflammatory processes [79]. AEA is also a ligand for the transient receptor potential vanilloid receptor type 1 (TRPV-1), which is expressed in peripheral sensory neurons and in the central nervous (CNS) system, where they have a role in regulating synaptic function [80].

Endocannabinoids other than 2-AG and AEA either do not bind orthosterically with CB1 or CB2 receptors or bind with much lower affinity; however, they still exhibit cannabimimetic activities and potentiate the activity of 2-AG and AEA, in a phenomenon called the ‘entourage effect’ [56, 81]. However, evidence suggests that the relationship between 2-AG and AEA and their congeners is much more nuanced than this, and other endocannabinoids have been reported to either serve as functional antagonists [81] or act via non-endocannabinoid pathways. For example, DHEA activates protein kinase A (PKA)/cAMP response element binding protein (CREB) pathways [82].

Little is known about the process of endocannabinoid transport across cell membranes, although a putative endocannabinoid cell membrane transporter has been implicated in the control of AEA and 2-AG transport and metabolism [83]. The hydrolysis of AEA releases ARA and ethanolamine and is principally achieved by the fatty acid amide hydrolase (FAAH) enzyme [84], although further yet to be identified proteins are likely involved in the process [61]. DHEA is also a substrate for FAAH hydrolysis to release DHA and ethanolamine [68], whereas the process of EPEA hydrolysis has yet to be identified. Unlike the ethanolamides, a variety of enzymes are responsible for the degradation of 2-AG to ARA and glycerol, with three serine hydrolases accounting for approximately 99% of hydrolysis in the brain [85]. Approximately 85% of 2-AG hydrolysis occurs via monoacylglycerol lipase (MAGL), which is co-localised with CB1 receptors in axon terminals [85]. ABHD6 and ABHD12 account for approximately 4 and 9% of brain 2-AG hydrolase activity, respectively, with ABHD6 located in post-synaptic neurons and ABHD12 is highly expressed in microglia [85]. 2-AG hydrolysis may also be catalysed by FAAH [86]. The pathways(s) of 2-DPG and 2-EPG hydrolysis are currently unknown.

In addition to the direct signalling roles of 2-AG and AEA, both are important intermediates in lipid metabolism. They act as precursor pools for ARA for the subsequent production of eicosanoids [87] and are also converted to further classes of bioactive mediators. 2-AG and AEA are substrates for COX-2, producing prostamides and prostaglandin glycerol esters, LOX producing hydroperoxy derivatives (HPETE) and CYP enzymes, producing hydroxy-eicosatetraenoic ethanolamide molecules (HETE-EA) or epoxy-eicosatrienoic acids (EET) [30, 88, 89]. 2-AG can also be phosphorylated by acyl glycerol kinase(s) to produce lysophosphatidic acid (LPA) [66], another important bioactive lipid [90]. Interestingly, COX-2 metabolites of 2-AG and AEA have been shown to have opposing effects to those of 2-AG and AEA themselves, suggesting a fine balance in the control of synaptic transmission between these lipid mediators and their oxygenated products [91].

The oxidative metabolism of DHA and EPA-derived endocannabinoids is beginning to be elucidated, but there is much that is currently unknown. Lipidomic screening has identified oxygenated products of DHEA generated from LOX and includes 10,17-dihydroxy-docosahexaenoyl ethanolamide (10,17-diHDoHE) and hydroxy-16(17)-epoxy-docosapentaenoyl ethanolamide (HEDPEA) [68]. These molecules exhibit anti-inflammatory and organ-protective effects in a mouse reperfusion second organ injury [68].

The multiple lipid mediators derived from ARA, DHA and EPA are summarised in Fig. 2, where is can be seen that the lipidome of ARA is the best characterised; however, analogous repertoires of mediators are likely produced from DHA and EPA and potentially other PUFAs. Recent developments in lipidomic analyses have greatly increased interest in the discovery, identification and elucidation of the multiple mediators derived from PUFA and endocannabinoids, but much more work is needed to fully develop understanding of their biological activities and the effects of changing dietary intake and subsequent phospholipid PUFA composition on their formation. The remainder of this review will summarise current evidence of the interplay between n-3 and n-6 LCPUFA and endocannabinoids in neuroinflammation, neurogenesis and brain ageing.

## Neuroinflammation

Neuroinflammation is the CNS process to restore damaged neurons and glia, with microglia and astrocytes the predominant effectors [92]. Activation of microglia initiates a rapid response involving migration, proliferation, and the release of cytokines and chemokines [93]. This is initially a protective response, but excess neuroinflammation may inhibit neuronal regeneration and if it becomes chronic play an important role in the pathogenesis of neurodegenerative diseases, such as Alzheimer’s disease (AD) and Parkinson’s disease (PD), by secreting cytotoxic proteins and reactive oxygen species [94].

In the healthy brain microglia display a “resting” phenotype responsible for continuous immune monitoring and surveillance and also play a key role in regulating neuronal plasticity via processes including synaptic pruning and neurogenesis [95]. Pathological conditions such as damage to neural cells causes the local “resting” microglia to respond by “activation” and rapidly change their phenotype and redirect their activity [96]. Depending on the type and extent of stimulation the expression of specific genes is induced tailoring the microglial phenotype towards either the classically activated (M1) pro-inflammatory phenotype or the alternatively activated (M2) anti-inflammatory phenotype [96], although the further M2a and M2c phenotypes have been identified based on the stimulus of induction [97].

Work by our laboratory and others has shown the elevated levels of n-3 PUFA reduces microglial activation and subsequent production of pro-inflammatory cytokines in a wide variety of models of neuroinflammation, such as amyotrophic lateral sclerosis [98], spinal cord injury [99, 100], ischaemia [101] and brain ageing [102]. Recent work has begun to explore the mechanisms behind these effects. DHA down-regulates the cell-surface expression of cluster of differentiation 14 (CD14) and Toll-like receptor 4 (TLR4) in lipopolysaccharide (LPS)-stimulated microglial cells [103]. CD14 is a glycosylphosphatidylinositol-linked protein and transduces the signal by associating with other partners, especially TLR4 [104].

N-3 PUFA supplementation also inhibits microglial activation by inhibiting nuclear translocation and secretion of high-mobility group box 1 (HMGB1) and HMGB1-mediated activation of TLR4/NF-κβ signalling pathways in a model of traumatic brain injury [105]. HMGB1 is a central component of the late inflammatory response and the translocation and secretion of HMGB1 are important steps in HMGB1-induced inflammation [106]. After release, HMGB1 binds to transmembrane TLR4 and activates the TLR4/NF-κB signalling pathway, ultimately leading to neuroinflammation [107]. In this study n-3 PUFA supplementation inhibited the translocation of NF-κB p65 from the cytosol to the nucleus, reduced NF-κB p65 expression and inhibited the expression of the TLR4/NF-κB signalling pathway-associated proteins.

Taken together these results suggest n-3 PUFAs regulate microglial activation at several stages; however, these effects could be mediated by the n-PUFA themselves or their respective SPM. For example, both DHA and NPD1 block production of cytokines by microglial cells in a variety of retinal and brain injury models [108, 109]. RvD1 and MaR1 down-regulate *in vitro* microglia activation [110], RvD2 inhibits LPS-induced increase of TLR4 in microglia [111], and RvE1 alters the inflammatory response and decreases microglial activation in several *in vivo* models [112, 113].

During neuroinflammation there is a general up-regulation of the activity of the endocannabinoid system, with predominantly anti-inflammatory effects [114]. However, studies looking at the role of endocannabinoids in neuroinflammation tend to focus on the role of CB2 receptors, as CB2 receptors are more abundant than CB1 on microglia [115] and CB2 receptor expression is increased in microglia and astrocytes during neuroinflammation [74], where they attenuate the release of cytokines from activated microglia [8]. Furthermore, microglia from CB2 receptor knock-out mice show a decrease in phagocytic activity and CB2 receptor antagonists reduce motility of microglia *in vitro* [116]. Furthermore, microglia in brain tissue from patients with Alzheimer’s disease (AD), multiple sclerosis and amyotrophic lateral sclerosis express CB2 receptors [115]. However, recent work suggests a more complex story, with the endocannabinoid system responsive to the M2 phenotype [117]. CB1 and CB2 receptors are down-regulated in M1 microglia, whereas the M2a and M2c microglia show phenotypic changes in the endocannabinoid machinery, such that M2a favours 2-AG synthesis and M2c favours AEA. A recent study also highlighted the role of endocannabinoids in microglia-neuron signalling [118]. Endocannabinoids were secreted through microglial extracellular membrane vesicles and these extracellular vesicles carry AEA on their surface, which stimulates CB1 receptors on neurons and inhibits presynaptic transmission.

In addition to microglia, astrocytes respond to CNS damage and disease via the process of “reactive astrogliosis” [119]. In this process astrocytes respond to and also produce a wide range of cytokines and inflammatory mediators and interact with an array of cell types, thereby mediating crosstalk between neuroinflammatory and neural systems [120]. Astrocytes also have regulatory roles in PUFA metabolism and endocannabinoid signalling and promote endocannabinoid crosstalk with other lipid mediators. Astrocytes are able to synthesise ARA and DHA from LA and ALA, respectively [121], although astrocytic DHA synthesis is much lower than brain DHA uptake and utilisation rates, suggesting astrocyte synthesis does not provide a major contribution [20]. Astrocytes highly express MAGL and mice with specific astrocytic MAGL deletion exhibit moderately increased 2-AG and reduced ARA levels and reduced PGE_2_ and pro-inflammatory cytokine levels upon LPS administration, indicating an important role for astrocytes in endocannabinoid signalling in neuroinflammation [122]. Furthermore, using an inducible knock-out system the metabolism of 2-AG was shown to be co-ordinately regulated by neurons and astrocytes and involved transcellular shuttling of lipid substrates, such as ARA and eicosanoids [123]. This astrocyte-neuronal crosstalk may provide an integrated regulation of 2-AG metabolism and prevent excessive CB1 receptor activation.

Taken together, these studies show n-3 PUFA and their SPMs, and 2-AG and AEA play important roles in the regulation of the neuroinflammatory responses of microglia and astrocytes. However, with a greater understanding of the mechanisms by which these lipid mediators interact with each other and with microglia, astrocytes and surrounding neurons it may be possible to develop effective approaches to regulating neuroinflammation via manipulation of dietary n-6 and n-3 PUFA intake.

## Learning, Memory and Synaptic Plasticity

N-3 PUFA supplementation benefits many aspects of learning and memory, and although a number of putative targets have been identified, the exact mechanisms underlying these effects are still unresolved [1]. A study by Pan and co-workers suggests that these positive effects may be dependent on modulation of the endocannabinoid system [124]. The spatial memory of rats treated with DHA significantly improved at lower doses (150 or 300 mg/kg/day), whereas at a higher level of intake (600 mg/kg/day) it was impaired. These *in vivo* dose-dependent effects were highly correlated with similar *in vitro* dose-dependent up-regulation of CB1 and TRPV-1 receptors in cultured hippocampal neurons. The authors concluded that CB1 and TRPV-1 may therefore be involved in positive effects of DHA supplementation on spatial memory, although further work is needed to confirm this.

Synaptic plasticity is a widespread CNS phenomenon that occurs at both excitatory and inhibitory synapses, where changes in synaptic efficacy and strength are induced in response to various stimuli, and this potentiation or depression is thought to underlie phenomena such as learning and memory [125]. The endocannabinoid system positively modulates many aspects of synaptic plasticity [126], and a recent elegant series of studies by Layė and co-workers shows the essential role of n-3 PUFA in these effects [13, 14, 127]. In the first of these studies, long-term n-3 PUFA deficiency prevented endocannabinoid-mediated long-term synaptic depression (LTD) in the prefrontal cortex and nucleus accumbens [13]. Cannabinoid receptors couple to G protein type Gi/o and activate signalling pathways [74], and in this study CB1 receptors were uncoupled from their G(i/o) proteins. In the follow-up studies, similar effects on other measures of endocannabinoid-dependent plasticity were also found in other brain regions, including the hypothalamus [14] and hippocampus [127]. In the hippocampus, loss of N-methyl-d-aspartate (NMDA) glutamate receptor-dependent LTP induced by n-3 PUFA deficiency was shown to be due to the ablation of endocannabinoid-mediated inhibitory LTD (iLTD) [127]. In the hippocampus LTP is gated by the process of heterosynaptic iLTD, which is dependent on the activation of CB1 receptors [80]. Overall, the role of n-PUFA regulation of the endocannabinoid system in learning, memory and synaptic plasticity appears more complex than simply the modulation of endocannabinoid levels, but also critically depends on modulating receptor function.

## Neurogenesis

Neurogenesis in the adult brain from precursor neural stem cells has been identified consistently in two regions, the subgranular layer of the hippocampal dentate gyrus and the subventricular zone (SVZ), where it has been reported in all mammals studied, including humans [128]. The hippocampus is essential for learning and memory formation and consolidation and also important in regulating aspects of emotion, fear, anxiety and stress [129]. However, the hippocampus is particularly vulnerable to neuroinflammation, ageing and neurodegeneration [129]; indeed ageing is the greatest negative regulator of hippocampal neurogenesis [130]. It is therefore interesting to note that hippocampal neurogenesis has been shown to increase following ischaemia [131], stroke [132] and seizures [133], where the increases may be considered an attempt by the brain at self-repair. Enhancing hippocampal neurogenesis may therefore offer a novel therapeutic approach in the treatment of brain ageing and neurodegeneration.

DHA and EPA treatment has consistently been shown to increase adult hippocampal neurogenesis across a range of animal models [134], also in neural stem cells, where DHA appears to promote neuronal differentiation [73]. Similarly, the endocannabinoid system is essential for adult neurogenesis in both the hippocampus [135, 136] and SVZ [137], although studies into the pro-neurogeneic effects of endocannabinoids in the dentate gyrus have produced conflicting results. For example, adult rats treated with the AEA analogue methanandamide have significantly decreased hippocampal neurogenesis, which is increased by CB1 antagonists [136]. However, chronic treatment with a synthetic endocannabinoid agonist increases adult hippocampal neurogenesis in rats [138], and CB1 receptor knock-out mice show significant reductions in neurogenesis in the dentate gyrus and SVZ [135]. Pharmacological blockade of DAGL and CB2 with specific antagonists inhibits the proliferation of neural stem cells and the proliferation of progenitor cells in young animals [137]. A similar response is seen with a FAAH inhibitor [139]. Overall, the effects of the endocannabinoid system on neurogenesis appear to be a fine balance of receptor activation.

Work in our laboratory is the first to explore the role of the endocannabinoid system in the pro-neurogeneic effects of DHA and EPA [73]. In this study, addition of DHA or EPA to neural stem cells induces opposing effects on cell fate, which are directed by different signalling pathways. Although both DHA and EPA significantly increase 2-AG levels, only EPA utilises endocannabinoid signalling pathways to increase proliferation. EPA increases proliferation via CB1/2 receptors, which activate the p38 mitogen-activated protein kinase (p38 MAPK) signalling pathway. DHA was found to decrease cell proliferation, consistent with induction of differentiation. It may be hypothesised that although 2-AG is increased by DHA, the effects may be mitigated and cell fate directed towards differentiation via alternative pathways, such as through conversion to DHEA [82]. Rashid and co-workers show that DHEA induces differentiation of neural stem cells via protein kinase A (PKA)/cAMP response element binding protein (CREB). It may therefore be that DHA and EPA direct cell fate via alternative pathways determined by the levels and types of mediators produced.

In addition, our study also identified a previously unrecognised role of the immune system in the effects of DHA and EPA [73]. DHA and EPA treatment of neural stem cells from interleukin-1β (IL-1β) knock-out mice induced effects quite distinct from the wild-type cells, whereby proliferation was increased by DHA and reduced by EPA. As p38 MAPK was not activated by DHA, this suggests alterative non-endocannabinoid pathways were behind the increases in proliferation.

## The Ageing Brain

Normal brain ageing is characterised by many detrimental changes, such as mitochondrial dysfunction and alterations in energy metabolism [140], damage to DNA [141], increased microglial activation [142] and increased oxidative stress [143]. The ageing brain is also prone to development of neurodegenerative diseases, such as AD and PD, but with the protracted pre-symptomatic stages it is hard to identify what are normal age-related changes and what are effects of undetected neurodegeneration [144].

Many epidemiological studies suggest positive associations between an elevated dietary intake of n-3 PUFA and the maintenance of cognitive function in old age [3]. However, the results of randomised controlled trials in this area have been mixed, although positive study outcomes with higher doses of DHA in particular in asymptomatic participants or those with very mild memory deficits suggest supplementation is most effective in the pre-symptomatic stage, prior to the onset of mild cognitive impairment or dementia [145–147].

Studies in both rodents and humans show that the endocannabinoid system is susceptible to age-related deficits [74]. For example, CB1 receptor levels decrease, along with the activity NAPE-PLD and DAGL [74]. Furthermore, decreases in DAGL, coupled with elevated MAGL, leads to specific decreases in 2-AG levels in the hippocampus of ageing mice [148]. Using mouse genetic CB1 receptor knock-out models, it is possible to mimic the effects of these age-related changes [74]. CB1 receptor deletion leads to an age-dependent acceleration of cognitive decline with accelerated hippocampal neuronal loss and increases aspects of neuroinflammation, such as reactive astrogliosis and microglial activation.

These studies suggest that the age-related decline of specific components of the endocannabinoid system accelerates key aspects of brain ageing; therefore, through the restoration or reversal of the age-related effects it may be able to decrease this decline. In addition to modulating the levels of 2-AG and AEA, expressions of CB1 receptors, TRPV-1 and PPARγ have all been shown to be responsive to n-3 PUFA treatment [79, 124], suggesting that n-3 PUFA may be able to mitigate or reverse some of these age-related losses. Furthermore, these positive effects on the endocannabinoid system may potentially contribute to some of the protective effects of n-3 PUFA observed in studies in ageing. However, much more research is required to develop our understanding of the mechanisms underlying these effects and the consequences for the endocannabinoid system to maximise the therapeutic potential of n-3 PUFA in brain protection and repair.

## Conclusions

Due to their fundamental nature, ARA, DHA, EPA and their mediators and the endocannabinoid system have wide-ranging effects across the CNS and recent evidence strongly indicates a complex interplay between them. The levels of phospholipid-bound ARA determine the levels of 2-AG and AEA, which in addition to their own biological activities act as reservoirs of ARA for subsequent eicosanoid production. Importantly, brain LCPUFA levels are responsive to dietary intake, and the n-6:n-3 PUFA ratio of the current Western diet may lead to increased neuroinflammation and also overstimulation of the endocannabinoid system.

Neuroinflammation is a key feature of brain ageing and neurodegeneration and the development of new therapeutic approaches is necessary. Epidemiological studies consistently show beneficial effects of an elevated intake of DHA and EPA; however, these observations have so far failed to lead to new treatments. Trials typically provide n-3 PUFA in the form of fish oils, mixed DHA and EPA preparations or separate DHA and EPA, with limited consideration of the background levels of n-6 PUFA. It is hoped that a greater understanding of the relationship among ARA, DHA, EPA and the endocannabinoid system will lead to advances in developing their therapeutic potential and ultimately lead to the development of more targeted treatment options for brain protection and repair.

## Abbreviations

2-AG: 2-Arachidonoylglycerol
2-AG-LPA: 2-Arachidonoylglycerol-lysophosphatidic acid
2-AG-LPI: 2-Arachidonoyl-lysophosphatidylinositol
2-DHG: 2-Docosahexaenoylglycerol
2-EET-EG: 2-Epoxy-eicosatrienoic acid glycerol
2-EPG: 2-Eicosapentaenoylglycerol
A-COX: Acetylated COX-2
ABHD4: α/β-Hydrolase domain containing 4
ABHD6: α/β-Hydrolase domain containing 6
ABHD12: α/β-Hydrolase domain containing 12
AdA: Adrenic acid
AEA: *N*-arachidonoylethanolamide (anandamide)
ARA: Arachidonic acid
AT: Aspirin-triggered
COX-2: Cyclooxygenase-2
CYP: Cytochrome P450 monooxygenase
DAGL: Diacylglycerol lipase
DGLA: Dihomo-γ-linolenic acid
DHA: Docosahexaenoic acid
DHEA: *N*-docosahexaenoylethanolamine (synaptamide)
DiHDoHE: Dihydroxy-docosahexaenoic acid
DiHDPE: Dihydroxy-docosapentaenoic acid
DiHEPE: Dihydroxy-eicosapentaenoic acid
DiHETE: Dihydroxy-eicosatetraenoic acid
DiHETrE: Dihydroxy-eicosatrienoic acid
DPA: Docosapentaenoic acid
eCB: Endocannabinoid
EDP: Epoxy-docosapentaenoic acid
EET: Epoxy-eicosatrienoic acid
EET-EA: Epoxy-eicosatrienoic acid ethanolamide
EETeTr: Epoxy-eicosatetraenoic acids
EFOX: Electrophilic fatty acid oxo-derivative
EPA: Eicosapentaenoic acid
EpDPE: Epoxy-docosapentaenoic acid
EPEA: *N*-eicosapentaenoylethanolamine
EpETE: Epoxy-eicosapentaenoic acid
EpETrE: Epoxy-eicosatrienoic acid
Epo: Epoxygenase
FAAH: Fatty acid amide hydrolase
GP-NAPE: Glycerophosphoarachidonoylethanolamide
HDoHE: Hydroxy-docosahexaenoic acid
HEDPEA: Hydroxy-epoxy-docosapentaenoylethanolamide
HEET-EA: Hydroxy-epoxy-eicosatrienoic acid ethanolamide
HEPE: Hydroxy-eicosapentaenoic acid
HETE: Hydroxy-eicosatetraenoic acid
HETE-EA: Hydroxy-eicosatetraenoic acid ethanolamide
HHTrE: Hydroxy-heptadecatrienoic acid
HpDoHE: Hydroperoxy-docosahexaenoic acid
HpEPE: Hydroperoxy-eicosapentaenoic acid
HpETE: Hydroperoxy-eicosatetraenoic acid
Hx: Hepoxilin
LCPUFA: Long-chain polyunsaturated fatty acid
LOX: Lipoxygenase
Lt: Leukotriene
LTD: Long-term depression
LTP: Long-term potentiation
Lx: Lipoxin
MAGL: Monoacylglycerol lipase
MaR: Maresin
(N)PD1: (Neuro)protection D1
NAPE-PLD: *N*-acyl phosphatidylethanolamine-selective phospholipase D
NArPE: *N*-arachidonoyl phosphatidylethanolamine
NAT: *N*-acyltransferase
oxo-EET: Oxo-eicosatetraenoic acid
PAEA: Phospho-anandamide
PD: Protectin
PDE: Phosphodiesterase
PE: Phosphatidylethanolamine
PGD: Prostaglandin D metabolite
PGE: Prostaglandin E metabolite
PGF: Prostaglandin F metabolite
PGI: Prostacyclin
PGS: Prostaglandin E, D or F or prostacyclin synthase
PI: Phosphatidylinositol
PLA_1_: Phospholipase A_1_
PLC: Phospholipase C
PLD: Phospholipase D
PPAR: Peroxisome proliferator-activated receptor
PTPN22: Protein tyrosine phosphatase 22
RvD: Resolvin D series
RvE: Resolvin E series
Trx: Trioxilin
Tx: Thromboxane
TXS: Thromboxane synthase
SDA: Stearidonic acid
SVZ: Subventricular zone
TRPV-1: Transient receptor potential vanilloid receptor type 1
ϖ-H: ϖ-hydrolase
